# 2,5-Bis[4-(dimethyl­amino)­phen­yl]-3,6-dimethyl­pyrazine

**DOI:** 10.1107/S160053681101748X

**Published:** 2011-05-14

**Authors:** Sebastian Moschel, Dieter Schollmeyer, Heiner Detert

**Affiliations:** a University Mainz, Duesbergweg 10-14, 55099 Mainz, Germany

## Abstract

The title compound, C_22_H_26_N_4_, was prepared from *p*-dimethyl­amino­propiophenone in six steps. The mol­ecule has no crystallographic symmetry. The dihedral angles between the pyrazine ring and the phenyl rings are 35.81 (6) and 37.11 (8)°. The dimethyl­amino groups are essentially planar (sum of the bond angles at N = 359.3 and 359.9°) and nearly coplanar with the adjacent aromatic ring [dihedral angles = 5.54 (11) and 7.40 (3)°]. This effect and the short aniline C—N bonds can be rationalised in terms of charge transfer from the amino groups to the central pyrazine ring.

## Related literature

The title compound was prepared as a fundamental chromophore and as an inter­mediate for the preparation of acidochromic dyes, see: Detert & Sugiono (2005 ▶); Schmitt *et al.* (2008 ▶); Nemkovich *et al.* (2010 ▶). Conjugated oligomers with a pyrazine center and lateral donors are solvatochromic probes, see: Collette & Harper (2003 ▶) and Schmitt *et al.* (2011 ▶). 2,5-Diphenyl­pyrazine shows inter­planar angles of about 21° (Pieterse *et al.*, 2000 ▶); due to steric hindrance these angles are opened up to 37–49° in the tetra­phenyl­pyrazine (Bartnik *et al.*, 1999 ▶). The planarization of terminal amino groups and short aniline C—N bonds due to strong electronic coupling has also been observed in 2,5-bis­(*p*-dimethyl­amino­styr­yl)pyrazine, see: Fischer *et al.* (2011 ▶).
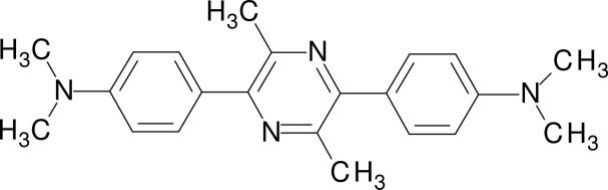


## Experimental

### 

#### Crystal data

C_22_H_26_N_4_
*M* *_r_* = 346.47
Triclinic, 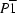

*a* = 9.459 (1) Å
*b* = 9.6368 (16) Å
*c* = 11.9661 (15) Å
α = 73.30 (1)°
β = 69.465 (11)°
γ = 74.446 (10)°
*V* = 961.2 (2) Å^3^
*Z* = 2
Cu *K*α radiation
μ = 0.56 mm^−1^
*T* = 193 K
0.40 × 0.20 × 0.05 mm

#### Data collection

Enraf–Nonius CAD-4 diffractometer
3876 measured reflections
3646 independent reflections
2922 reflections with *I* > 2σ(*I*)
*R*_int_ = 0.027
3 standard reflections every 60 min  intensity decay: 2%

#### Refinement

*R*[*F* ^2^ > 2σ(*F* ^2^)] = 0.063
*wR*(*F* ^2^) = 0.218
*S* = 1.07
3646 reflections
242 parameters
H-atom parameters constrained
Δρ_max_ = 0.43 e Å^−3^
Δρ_min_ = −0.42 e Å^−3^

### 

Data collection: *CAD-4 Software* (Enraf–Nonius, 1989 ▶); cell refinement: *CAD-4 Software*; data reduction: *CORINC* (Dräger & Gattow, 1971 ▶); program(s) used to solve structure: *SIR97* (Altomare *et al.*, 1999 ▶); program(s) used to refine structure: *SHELXL97* (Sheldrick, 2008 ▶); molecular graphics: *PLATON* (Spek, 2009 ▶); software used to prepare material for publication: *PLATON*.

## Supplementary Material

Crystal structure: contains datablocks I, global. DOI: 10.1107/S160053681101748X/bt5544sup1.cif

Structure factors: contains datablocks I. DOI: 10.1107/S160053681101748X/bt5544Isup2.hkl

Supplementary material file. DOI: 10.1107/S160053681101748X/bt5544Isup3.cdx

Supplementary material file. DOI: 10.1107/S160053681101748X/bt5544Isup4.cml

Additional supplementary materials:  crystallographic information; 3D view; checkCIF report

## Figures and Tables

**Fig. 1.**
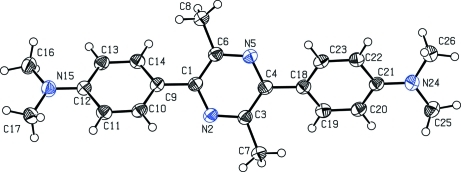
View of compound I. Displacement ellipsoids are drawn at the 50% probability level.

## supplementary crystallographic information

### Comment

The title compound was prepared as a fundamental chromophore and as an intermediate for the preparation of acidochromic dyes, see: Detert & Sugiono (2005); Schmitt *et al.* (2008); and Nemkovich *et al.* (2010). The synthesis started with the α-bromination of *p*-dimethylaminopropiophenone, nucleophilic bromine-azide exchange, reaction of the azide with triphenylphosphine followed by hydrolytic cleavage of the phosphorane imine and *in situ* condensation of the α-aminoketone to a dihydropyrazine that was directly air- oxidized to the title compound.

Though the molecular formula implies a center of inversion, the conformation of the title compound in the crystals is not centrosymmetric. Twists around the biaryl bonds with dihedral angles of -142.6 (2)° for N2—C1—C9—C14 and -144.41 (19)° for N5—C4—C18—C19 result in a helical conformation of the molecule. The dimethylamino groups are slightly twisted out of the plane of the benzene rings, with 9° or less, these deviations from coplanarity of these units and the adjacent phenyl rings are only small. The amino groups are planar, the sums of the bond angles at N15 amounts to 359.3° and to 359.9° at N24. This and the short aniline C—N bonds of 1.376 (2)Å for C12—N15 and C21—N24 prove a strong electronic coupling between the amino group and the pyrazine ring, similar to the related 2,5-bis(*p*-dimethylaminostyryl)pyrazine (Fischer *et al.*, 2011).

### Experimental

Synthesis: 810 mg (3.2 mmol) of 2-bromo-1-(4-(dimethylamino)-phenyl)-propan-1-one were dissolved in 50 ml of methanol and NaN_3_ (325 mg,5 mmol) was added. After stirring over night at room temperature the solvent was evaporated under vacuum and the residue was diluted with water. The aqueous solution was extracted three times with dichloromethane. The organic layers were dried (MgSO_4_) and the solvent was removed. Yield: 650 mg (3 mmol, 95%) of the crude 2-azido-1-(4-(dimethylamino)-phenyl)-propan-1-one as a yellow oil which was used without further purification. To a solution of 59 mg (0.27 mmol) 2-azido-1-(4-(dimethylamino)-phenyl)-propan-1-one in dry THF under nitrogen was added of triphenylphosphine (85 mg,0.32 mmol). The solution was stirred vigorously at room temperature until the nitrogen evolution ceased. 0.5 ml of water were added and stirring was continued overnight. The solvent was evaporated and the residue was dissolved in methanol. The reaction mixture was heated up to 333 K and air was passed through the solution for 5 h. Upon cooling the solution to 252 K, pure 2,5-bis(4-(dimethylamino)-phenyl)-3,6-dimethylpyrazine precipitated as a pale yellow solid. Yield: 20 mg (0.06 mmol, 43%). Red crystals were obtained by slow evaporation of a solution in methanol/dichloromethane. *M*.p.: 484 K.

### Refinement

Hydrogen atoms attached to carbons were placed at calculated positions with C—H = 0.95 Å (aromatic) or 0.98 Å (methyl groups). All H atoms were refined in the riding-model approximation with isotropic displacement parameters  set at 1.2–1.5 times of the *U*_eq_ of the parent atom.

### Crystal data

**Table:**

C_22_H_26_N_4_	*Z* = 2
*M**_r_* = 346.47	*F*(000) = 372
Triclinic, *P*1	*D*_x_ = 1.197 Mg m^−^^3^
Hall symbol: -P 1	Melting point: 484 K
*a* = 9.459 (1) Å	Cu *K*α radiation, λ = 1.54178 Å
*b* = 9.6368 (16) Å	Cell parameters from 25 reflections
*c* = 11.9661 (15) Å	θ = 60–69°
α = 73.30 (1)°	µ = 0.56 mm^−^^1^
β = 69.465 (11)°	*T* = 193 K
γ = 74.446 (10)°	Plate, yellow
*V* = 961.2 (2) Å^3^	0.40 × 0.20 × 0.05 mm

### Data collection

**Table:**

Enraf–Nonius CAD-4 diffractometer	*R*_int_ = 0.027
Radiation source: rotating anode	θ_max_ = 70.0°, θ_min_ = 4.0°
graphite	*h* = −11→11
ω/2θ scans	*k* = −11→0
3876 measured reflections	*l* = −14→13
3646 independent reflections	3 standard reflections every 60 min
2922 reflections with *I* > 2σ(*I*)	intensity decay: 2%

### Refinement

**Table:**

Refinement on *F*^2^	Secondary atom site location: difference Fourier map
Least-squares matrix: full	Hydrogen site location: inferred from neighbouring sites
*R*[*F*^2^ > 2σ(*F*^2^)] = 0.063	H-atom parameters constrained
*wR*(*F*^2^) = 0.218	*w* = 1/[σ^2^(*F*_o_^2^) + (0.1432*P*)^2^ + 0.2122*P*] where *P* = (*F*_o_^2^ + 2*F*_c_^2^)/3
*S* = 1.07	(Δ/σ)_max_ < 0.001
3646 reflections	Δρ_max_ = 0.43 e Å^−^^3^
242 parameters	Δρ_min_ = −0.42 e Å^−^^3^
0 restraints	Extinction correction: *SHELXL97* (Sheldrick, 2008), Fc^*^=kFc[1+0.001xFc^2^λ^3^/sin(2θ)]^-1/4^
Primary atom site location: structure-invariant direct methods	Extinction coefficient: 0.012 (2)

### Special details

**Table:**

Experimental. ^1^H-NMR (CDCl_3_): δ = 7.55 (d, J = 8.7 Hz, 4 H, 2-H, 6-H phenyl); 6.79 (br. d, J = 8.8 Hz, 4 H, 3-H, 5-H, phenyl); 3.03 (s, 12 H, N-CH_3_); 2.64 (s, 6 H, pyrazin-CH_3_). ^13^C-NMR (CDCl_3_): δ = 150.3, 150.1, 147.5 (C-2, C-3 pyrazine, C-1 phenyl), 130.5, 127.9 (C-2, C-3, C-5, C-6 phenyl), 112.9, 41.2 (N(CH_3_)_2_), 23.3 (CH_3_). FD-MS : 346.5 (100%) [M+] IR (ATR): ν = 2918.7, 1607.4, 1528.3, 1438.6, 1389.5, 1359.6, 1231.2, 1194.7, 1161.9, 943.0, 818.6, 785.9, 674.0 cm^-1^.
Geometry. All e.s.d.'s (except the e.s.d. in the dihedral angle between two l.s. planes) are estimated using the full covariance matrix. The cell e.s.d.'s are taken into account individually in the estimation of e.s.d.'s in distances, angles and torsion angles; correlations between e.s.d.'s in cell parameters are only used when they are defined by crystal symmetry. An approximate (isotropic) treatment of cell e.s.d.'s is used for estimating e.s.d.'s involving l.s. planes.
Refinement. Refinement of *F*^2^ against ALL reflections. The weighted *R*-factor *wR* and goodness of fit *S* are based on *F*^2^, conventional *R*-factors *R* are based on *F*, with *F* set to zero for negative *F*^2^. The threshold expression of *F*^2^ > σ(*F*^2^) is used only for calculating *R*-factors(gt) *etc*. and is not relevant to the choice of reflections for refinement. *R*-factors based on *F*^2^ are statistically about twice as large as those based on *F*, and *R*- factors based on ALL data will be even larger.

### Fractional atomic coordinates and isotropic or equivalent isotropic displacement parameters (Å^2^)

**Table:**

	*x*	*y*	*z*	*U*_iso_*/*U*_eq_	
C1	0.4300 (2)	0.7610 (2)	0.62142 (18)	0.0308 (4)	
N2	0.55091 (18)	0.65153 (18)	0.59277 (15)	0.0339 (4)	
C3	0.6186 (2)	0.6432 (2)	0.47567 (18)	0.0335 (5)	
C4	0.5629 (2)	0.7449 (2)	0.38283 (18)	0.0321 (5)	
N5	0.44665 (18)	0.85760 (18)	0.41135 (15)	0.0333 (4)	
C6	0.3811 (2)	0.8678 (2)	0.52829 (18)	0.0328 (5)	
C7	0.7572 (2)	0.5220 (2)	0.45446 (19)	0.0434 (6)	
H7A	0.8089	0.5052	0.5165	0.065*	
H7B	0.8282	0.5505	0.3731	0.065*	
H7C	0.7249	0.4312	0.4599	0.065*	
C8	0.2599 (2)	1.0036 (2)	0.54900 (19)	0.0407 (5)	
H8A	0.1592	0.9828	0.5607	0.061*	
H8B	0.2831	1.0845	0.4779	0.061*	
H8C	0.2586	1.0316	0.6219	0.061*	
C9	0.3625 (2)	0.7599 (2)	0.75399 (18)	0.0313 (5)	
C10	0.4576 (2)	0.7140 (2)	0.82934 (18)	0.0343 (5)	
H10	0.5649	0.6846	0.7941	0.041*	
C11	0.4019 (2)	0.7099 (2)	0.95281 (18)	0.0345 (5)	
H11	0.4708	0.6781	1.0008	0.041*	
C12	0.2435 (2)	0.7525 (2)	1.00883 (17)	0.0307 (5)	
C13	0.1460 (2)	0.7930 (2)	0.93438 (18)	0.0331 (5)	
H13	0.0380	0.8179	0.9697	0.040*	
C14	0.2057 (2)	0.7971 (2)	0.81013 (18)	0.0327 (5)	
H14	0.1374	0.8263	0.7618	0.039*	
N15	0.18654 (19)	0.7556 (2)	1.13101 (15)	0.0389 (5)	
C16	0.0227 (2)	0.7857 (3)	1.18829 (19)	0.0425 (5)	
H16A	−0.0203	0.8861	1.1522	0.064*	
H16B	0.0019	0.7762	1.2761	0.064*	
H16C	−0.0249	0.7152	1.1755	0.064*	
C17	0.2880 (3)	0.7075 (3)	1.20702 (19)	0.0435 (5)	
H17A	0.3251	0.6008	1.2163	0.065*	
H17B	0.2320	0.7306	1.2875	0.065*	
H17C	0.3756	0.7584	1.1687	0.065*	
C18	0.6290 (2)	0.7442 (2)	0.25089 (18)	0.0313 (5)	
C19	0.6822 (2)	0.6145 (2)	0.20663 (18)	0.0341 (5)	
H19	0.6779	0.5222	0.2629	0.041*	
C20	0.7408 (2)	0.6174 (2)	0.08291 (18)	0.0339 (5)	
H20	0.7764	0.5270	0.0561	0.041*	
C21	0.7490 (2)	0.7497 (2)	−0.00351 (17)	0.0310 (5)	
C22	0.6928 (2)	0.8808 (2)	0.04087 (18)	0.0331 (5)	
H22	0.6945	0.9734	−0.0152	0.040*	
C23	0.6352 (2)	0.8763 (2)	0.16485 (17)	0.0317 (5)	
H23	0.5987	0.9664	0.1921	0.038*	
N24	0.8089 (2)	0.75325 (19)	−0.12682 (15)	0.0399 (5)	
C25	0.8785 (3)	0.6186 (2)	−0.1704 (2)	0.0449 (6)	
H25A	0.9618	0.5652	−0.1348	0.067*	
H25B	0.9200	0.6421	−0.2597	0.067*	
H25C	0.8010	0.5570	−0.1465	0.067*	
C26	0.7970 (3)	0.8908 (2)	−0.21529 (19)	0.0436 (5)	
H26A	0.6886	0.9379	−0.2025	0.065*	
H26B	0.8410	0.8716	−0.2979	0.065*	
H26C	0.8532	0.9562	−0.2054	0.065*	

### Atomic displacement parameters (Å^2^)

**Table:**

	*U*^11^	*U*^22^	*U*^33^	*U*^12^	*U*^13^	*U*^23^
C1	0.0270 (9)	0.0304 (10)	0.0347 (10)	−0.0053 (7)	−0.0072 (8)	−0.0089 (8)
N2	0.0298 (8)	0.0339 (9)	0.0336 (9)	−0.0026 (7)	−0.0059 (7)	−0.0080 (7)
C3	0.0294 (9)	0.0339 (10)	0.0331 (10)	−0.0039 (8)	−0.0054 (8)	−0.0074 (8)
C4	0.0279 (9)	0.0323 (10)	0.0359 (10)	−0.0058 (8)	−0.0081 (8)	−0.0086 (8)
N5	0.0293 (8)	0.0345 (9)	0.0344 (9)	−0.0024 (7)	−0.0086 (7)	−0.0090 (7)
C6	0.0286 (9)	0.0340 (10)	0.0354 (10)	−0.0036 (8)	−0.0092 (8)	−0.0093 (8)
C7	0.0392 (11)	0.0414 (12)	0.0367 (11)	0.0079 (9)	−0.0084 (9)	−0.0071 (9)
C8	0.0383 (11)	0.0385 (11)	0.0386 (11)	0.0019 (9)	−0.0085 (9)	−0.0101 (9)
C9	0.0299 (9)	0.0313 (10)	0.0331 (10)	−0.0047 (8)	−0.0082 (8)	−0.0099 (8)
C10	0.0243 (9)	0.0371 (11)	0.0411 (11)	−0.0020 (8)	−0.0092 (8)	−0.0122 (8)
C11	0.0285 (9)	0.0395 (11)	0.0393 (11)	−0.0049 (8)	−0.0146 (8)	−0.0097 (8)
C12	0.0306 (10)	0.0304 (10)	0.0331 (10)	−0.0073 (8)	−0.0095 (8)	−0.0083 (8)
C13	0.0238 (9)	0.0378 (11)	0.0371 (10)	−0.0036 (7)	−0.0083 (7)	−0.0104 (8)
C14	0.0271 (9)	0.0380 (11)	0.0366 (10)	−0.0060 (8)	−0.0125 (8)	−0.0095 (8)
N15	0.0325 (9)	0.0514 (11)	0.0335 (9)	−0.0063 (8)	−0.0100 (7)	−0.0111 (8)
C16	0.0375 (11)	0.0494 (13)	0.0371 (11)	−0.0048 (9)	−0.0052 (9)	−0.0143 (9)
C17	0.0486 (12)	0.0466 (13)	0.0378 (11)	−0.0036 (10)	−0.0191 (9)	−0.0097 (9)
C18	0.0254 (9)	0.0346 (10)	0.0352 (10)	−0.0055 (8)	−0.0089 (8)	−0.0096 (8)
C19	0.0323 (10)	0.0326 (10)	0.0382 (11)	−0.0050 (8)	−0.0116 (8)	−0.0081 (8)
C20	0.0318 (10)	0.0316 (10)	0.0405 (11)	−0.0039 (8)	−0.0110 (8)	−0.0123 (8)
C21	0.0249 (9)	0.0342 (11)	0.0365 (10)	−0.0033 (8)	−0.0111 (8)	−0.0113 (8)
C22	0.0323 (10)	0.0313 (10)	0.0357 (10)	−0.0056 (8)	−0.0104 (8)	−0.0071 (8)
C23	0.0281 (9)	0.0290 (10)	0.0392 (10)	−0.0038 (7)	−0.0093 (8)	−0.0115 (8)
N24	0.0459 (10)	0.0381 (10)	0.0343 (10)	−0.0021 (8)	−0.0112 (8)	−0.0120 (8)
C25	0.0479 (12)	0.0444 (12)	0.0426 (12)	0.0013 (10)	−0.0130 (10)	−0.0194 (10)
C26	0.0465 (12)	0.0432 (12)	0.0375 (11)	−0.0042 (10)	−0.0119 (9)	−0.0079 (9)

### Geometric parameters (Å, °)

**Table:**

C1—N2	1.350 (2)	N15—C16	1.444 (3)
C1—C6	1.401 (3)	N15—C17	1.447 (3)
C1—C9	1.486 (3)	C16—H16A	0.9800
N2—C3	1.337 (2)	C16—H16B	0.9800
C3—C4	1.407 (3)	C16—H16C	0.9800
C3—C7	1.504 (3)	C17—H17A	0.9800
C4—N5	1.347 (2)	C17—H17B	0.9800
C4—C18	1.481 (3)	C17—H17C	0.9800
N5—C6	1.338 (2)	C18—C23	1.388 (3)
C6—C8	1.507 (3)	C18—C19	1.398 (3)
C7—H7A	0.9800	C19—C20	1.382 (3)
C7—H7B	0.9800	C19—H19	0.9500
C7—H7C	0.9800	C20—C21	1.393 (3)
C8—H8A	0.9800	C20—H20	0.9500
C8—H8B	0.9800	C21—N24	1.376 (2)
C8—H8C	0.9800	C21—C22	1.410 (3)
C9—C14	1.391 (3)	C22—C23	1.381 (3)
C9—C10	1.395 (3)	C22—H22	0.9500
C10—C11	1.376 (3)	C23—H23	0.9500
C10—H10	0.9500	N24—C25	1.442 (3)
C11—C12	1.410 (3)	N24—C26	1.445 (3)
C11—H11	0.9500	C25—H25A	0.9800
C12—N15	1.376 (2)	C25—H25B	0.9800
C12—C13	1.407 (3)	C25—H25C	0.9800
C13—C14	1.386 (3)	C26—H26A	0.9800
C13—H13	0.9500	C26—H26B	0.9800
C14—H14	0.9500	C26—H26C	0.9800
			
N2—C1—C6	119.83 (17)	C16—N15—C17	118.93 (17)
N2—C1—C9	115.15 (17)	N15—C16—H16A	109.5
C6—C1—C9	124.97 (17)	N15—C16—H16B	109.5
C3—N2—C1	119.37 (17)	H16A—C16—H16B	109.5
N2—C3—C4	120.69 (18)	N15—C16—H16C	109.5
N2—C3—C7	114.53 (17)	H16A—C16—H16C	109.5
C4—C3—C7	124.75 (17)	H16B—C16—H16C	109.5
N5—C4—C3	119.63 (18)	N15—C17—H17A	109.5
N5—C4—C18	115.47 (17)	N15—C17—H17B	109.5
C3—C4—C18	124.79 (17)	H17A—C17—H17B	109.5
C6—N5—C4	119.54 (17)	N15—C17—H17C	109.5
N5—C6—C1	120.72 (18)	H17A—C17—H17C	109.5
N5—C6—C8	115.08 (17)	H17B—C17—H17C	109.5
C1—C6—C8	124.14 (18)	C23—C18—C19	117.00 (18)
C3—C7—H7A	109.5	C23—C18—C4	120.18 (17)
C3—C7—H7B	109.5	C19—C18—C4	122.78 (18)
H7A—C7—H7B	109.5	C20—C19—C18	121.52 (18)
C3—C7—H7C	109.5	C20—C19—H19	119.2
H7A—C7—H7C	109.5	C18—C19—H19	119.2
H7B—C7—H7C	109.5	C19—C20—C21	121.55 (18)
C6—C8—H8A	109.5	C19—C20—H20	119.2
C6—C8—H8B	109.5	C21—C20—H20	119.2
H8A—C8—H8B	109.5	N24—C21—C20	121.82 (18)
C6—C8—H8C	109.5	N24—C21—C22	121.17 (18)
H8A—C8—H8C	109.5	C20—C21—C22	117.01 (18)
H8B—C8—H8C	109.5	C23—C22—C21	120.81 (18)
C14—C9—C10	116.81 (18)	C23—C22—H22	119.6
C14—C9—C1	123.42 (17)	C21—C22—H22	119.6
C10—C9—C1	119.72 (17)	C22—C23—C18	122.09 (18)
C11—C10—C9	122.45 (17)	C22—C23—H23	119.0
C11—C10—H10	118.8	C18—C23—H23	119.0
C9—C10—H10	118.8	C21—N24—C25	120.54 (17)
C10—C11—C12	120.62 (17)	C21—N24—C26	120.83 (17)
C10—C11—H11	119.7	C25—N24—C26	118.57 (17)
C12—C11—H11	119.7	N24—C25—H25A	109.5
N15—C12—C13	121.30 (17)	N24—C25—H25B	109.5
N15—C12—C11	121.43 (17)	H25A—C25—H25B	109.5
C13—C12—C11	117.27 (17)	N24—C25—H25C	109.5
C14—C13—C12	120.70 (17)	H25A—C25—H25C	109.5
C14—C13—H13	119.6	H25B—C25—H25C	109.5
C12—C13—H13	119.6	N24—C26—H26A	109.5
C13—C14—C9	122.06 (17)	N24—C26—H26B	109.5
C13—C14—H14	119.0	H26A—C26—H26B	109.5
C9—C14—H14	119.0	N24—C26—H26C	109.5
C12—N15—C16	120.04 (16)	H26A—C26—H26C	109.5
C12—N15—C17	120.38 (17)	H26B—C26—H26C	109.5
			
C6—C1—N2—C3	−2.7 (3)	C11—C12—C13—C14	−2.9 (3)
C9—C1—N2—C3	179.60 (17)	C12—C13—C14—C9	0.9 (3)
C1—N2—C3—C4	−1.5 (3)	C10—C9—C14—C13	1.7 (3)
C1—N2—C3—C7	176.75 (18)	C1—C9—C14—C13	179.07 (18)
N2—C3—C4—N5	4.4 (3)	C13—C12—N15—C16	6.7 (3)
C7—C3—C4—N5	−173.64 (19)	C11—C12—N15—C16	−173.95 (19)
N2—C3—C4—C18	−179.55 (18)	C13—C12—N15—C17	177.36 (18)
C7—C3—C4—C18	2.4 (3)	C11—C12—N15—C17	−3.3 (3)
C3—C4—N5—C6	−2.9 (3)	N5—C4—C18—C23	33.3 (3)
C18—C4—N5—C6	−179.32 (17)	C3—C4—C18—C23	−142.9 (2)
C4—N5—C6—C1	−1.3 (3)	N5—C4—C18—C19	−144.41 (19)
C4—N5—C6—C8	175.93 (17)	C3—C4—C18—C19	39.4 (3)
N2—C1—C6—N5	4.2 (3)	C23—C18—C19—C20	1.1 (3)
C9—C1—C6—N5	−178.35 (18)	C4—C18—C19—C20	178.86 (17)
N2—C1—C6—C8	−172.74 (18)	C18—C19—C20—C21	−0.3 (3)
C9—C1—C6—C8	4.7 (3)	C19—C20—C21—N24	179.26 (17)
N2—C1—C9—C14	−142.6 (2)	C19—C20—C21—C22	−0.8 (3)
C6—C1—C9—C14	39.9 (3)	N24—C21—C22—C23	−178.92 (17)
N2—C1—C9—C10	34.8 (3)	C20—C21—C22—C23	1.2 (3)
C6—C1—C9—C10	−142.8 (2)	C21—C22—C23—C18	−0.4 (3)
C14—C9—C10—C11	−2.2 (3)	C19—C18—C23—C22	−0.8 (3)
C1—C9—C10—C11	−179.68 (18)	C4—C18—C23—C22	−178.57 (17)
C9—C10—C11—C12	0.1 (3)	C20—C21—N24—C25	−5.7 (3)
C10—C11—C12—N15	−176.91 (18)	C22—C21—N24—C25	174.39 (18)
C10—C11—C12—C13	2.5 (3)	C20—C21—N24—C26	171.36 (18)
N15—C12—C13—C14	176.43 (18)	C22—C21—N24—C26	−8.5 (3)
